# The complete mitochondrial genome of White-cheeked macaque (*Macaca leucogenys*)

**DOI:** 10.1080/23802359.2016.1172039

**Published:** 2016-06-20

**Authors:** Wei Hou, Sanxu Liu, Juan Jiang, Zhenxin Fan, Pengfei Fan, Jing Li

**Affiliations:** a Key Laboratory of Bio-Resources and Eco-Environment (Ministry of Education), College of Life Sciences, Sichuan University, Chengdu, PR China;; b School of Life Science, Sun yat-Sen University, Guangzhou, PR China

**Keywords:** Genome structure, *Macaca leucogenys*, mitochondrial genome, phylogenetic tree

## Abstract

White-cheeked macaque is a newly described species in genus *Macaca*. Here, the complete mitochondrial genome was firstly determined, which was deposited in Genbank with accession number KU564271. The length of the mitogenome is 16,494 base pairs (bp). It includes 13 protein-coding genes (PCGs), 22 transfer RNA (tRNA) genes, two ribosomal RNA (rRNA) genes and a control region. We used 12 mitochondrion genes of 15 species to constructed phylogenetic tree with three methods. The study will provide useful data for further studies on phylogenetic relationships as well as population structure, biodiversity conservation and conservation genetics.

The white-cheeked macaque (*Macaca leucogenys*) was described as a new macaque species in Medog, southeastern Tibet, China (Li et al. 2015). Little studies have been done on molecule biology and genetics of *M. leucogenys*. Here, we sequenced the complete mitochondrion genome of *M. leucogenys* and 12 genes of 14 species were used to construct the phylogenetic tree, which will not only facilitate the phylogenetic estimation but also provide valuable data for studies of population structure and conservation genetics.

The specimen was collected from Medog, Tibet, China, which is stored in Dali University, Yunnan province and preserved at low temperature (−20 °C) with the accession number is XKL. The total genomic DNA was extracted with the method of standard phenol/chloroform (Sambrook & Russell 2001). The complete mitogenome of *M. leucogenys* is 16,494 bp, which was deposited in the Genbank with the accession number KU564271. It contains 13 protein-coding genes (PCGs), 22 transfer RNA genes, two ribosomal RNA genes and a control region. The base composition of complete mitochondrial genome is 31.44% A, 25.26% T, 30.08% C and 13.21% G. The content of A + T is 56.71%, which similar with other vertebrate mitochondrial genome (Zhang et al. 2003).

The PCGs regions of the *M. leucogenys* mitogenomes were consistent with those of other cercopithecidae mitogenomes (Boore 1999; Xing et al. 2007). Among the 13 PCGs, all the PCGs were located on H strand except *ND6* genes, which was encoded on L strand. The majority of the genes were initiated with ATG. Three genes were initiated with ATN. However, *ND1* used infrequently GTG as the start codon. Five PCGs used the standard codon TAA as stop codon, while *ND2*, *COXI* and *CYTB* used TAN as their incomplete stop codon. Single T was used as incomplete stop codon in *ND1*, *COXIII*, *ND3*, *ND4* and *ND6*.

Macaca *leucogenys* contained the typical domains of 22 tRNA genes, interspersing in the genome and ranging in size from 59 bp to 75 bp, in line with what have been found in most cercopithecidae (Juhling et al. 2012). The repeat region is longer than the *M. thibetana*. As the same as other cercopithecidae species, eight tRNA encoded on the L strand, while the other 14 encoded on H strand. Besides, we found that *tRNA-Glu* gene in *M. leucogenys* is longer than the other species (i.e. *M. thibetana*, *M. mulatta*, etc.). A control gene (D-loop) is 1065 bp with the typical gene order in vertebrates.

Macaca *leucogenys* is more similar to the silenus–sylvanus group, rather than the potential sympatric macaque species based on the structure of male external genitalia and morphology (i.e. *M. assamensis* and *M. mulatta*) (Li et al. 2009; Jiang et al. 2016). Phylogenetic analysis based on 12 common protein sequences except *ND6* in 15 species. Three methods (BI/MP/NJ) were used to constructed the phylogenetic tree (Figure 1), which confirmed that *M. leucogenys* as a member of genus *Macaca*. Macaca *leucogenys* is most closely to the species *M. assamensis* and *M. thibetana* analyzed from the tree. It provides useful data for further study on evolution of *M. leucogenys.*

**Figure 1.**
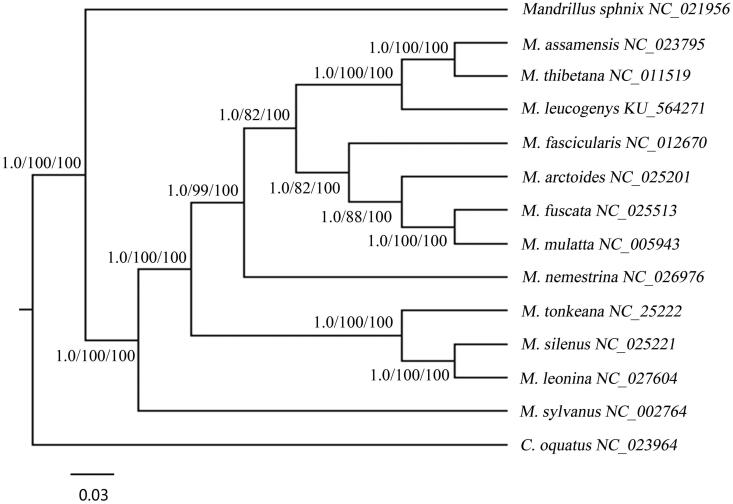
Three methods were used to construct the phylogenetic tree, Bayes tree, maximum parsimony method and neighbour-joining method, which constructed by MrBayes (MrBayes Inc., New York, NY), PAUP4.0 (Sinauer Associates, Sunderland, MA) and MEGA5.2 (MEGA Inc., Englewood, NJ) individually. *Macaca leucogenys* is closely to *Macaca assamensis* and *Macaca thibetana*. *Cerecocebus oquatus* species was set as the outgroup.
